# Hyperfine adjustment of flexible pore-surface pockets enables smart recognition of gas size and quadrupole moment†
†Electronic supplementary information (ESI) available: Experimental section, PXRD patterns, crystallographic tables and characterization details. CCDC 1561837–1561845. For ESI and crystallographic data in CIF or other electronic format see DOI: 10.1039/c7sc03067c



**DOI:** 10.1039/c7sc03067c

**Published:** 2017-09-11

**Authors:** Chun-Ting He, Zi-Ming Ye, Yan-Tong Xu, Dong-Dong Zhou, Hao-Long Zhou, Da Chen, Jie-Peng Zhang, Xiao-Ming Chen

**Affiliations:** a MOE Key Laboratory of Bioinorganic and Synthetic Chemistry , School of Chemistry , Sun Yat-Sen University , Guangzhou 510275 , China . Email: zhangjp7@mail.sysu.edu.cn

## Abstract

The pore size and framework flexibility of hosts are of vital importance for molecular recognition and related applications, but accurate control of these parameters is very challenging. We use the slight difference of metal ion size to achieve continuous hundredth-nanometer pore-size adjustments and drastic flexibility modulations in an ultramicroporous metal–organic framework, giving controllable N_2_ adsorption isotherm steps, unprecedented/reversed loading-dependence of H_2_ adsorption enthalpy, quadrupole-moment sieving of C_2_H_2_/CO_2_, and an exceptionally high working capacity for C_2_H_2_ storage under practical conditions (98 times that of an empty cylinder). *In situ* single-crystal X-ray diffraction measurements and multilevel computational simulations revealed the importance of pore-surface pockets, which utilize their size and electrostatic potential to smartly recognize the molecular sizes and quadruple moments of gas molecules to control their accessibility to the strongest adsorption sites.

## Abstract 2

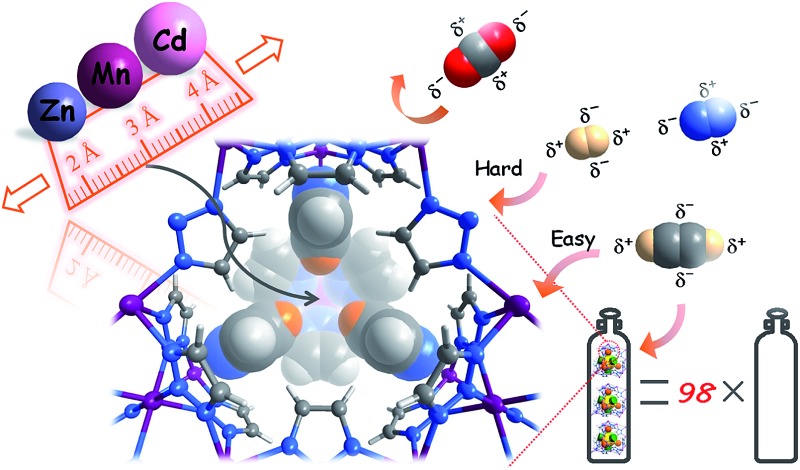
Continuous pore-size adjustments are achieved in a series of ultramicroporous MOFs, giving flexible pore-surface pockets for the smart recognition of highly similar gases and high gas separation/storage performances.

## Introduction

Smart materials exhibiting adaptive responses or recognition to guest molecules are of paramount importance for applications in high-tech areas.^1–3^ Size matching between the host and guest is the most important aspect of molecular recognition.^4–6^ Generally, the host size is modified by changing the number of repeating building units, which can achieve size intervals as small as *ca.* 0.1–0.3 nm (*e.g.*, cyclodextrins 0.14–0.21 nm,^2^ cucurbit[*n*]urils 0.14–0.26 nm,^7^ and zeolites 0.1 nm (ref. 8)), *i.e.*, the diameter of an atom. Biomacromolecules such as proteins can precisely fit complicated molecules with additional help from shape matching, either through the lock-and-key or induced-fit (structural deformation) mechanisms.^9,10^ However, the available strategies for molecular recognition can hardly work for gas molecules with very small sizes and simple shapes.

Porous coordination polymers (PCPs), also known as metal–organic frameworks (MOFs), have been demonstrated as a promising type of host materials, mainly because of their readily tunable pore sizes/shapes^11,12^ and notable framework flexibilities.^13–15^ For instance, the channel size of the MOF-74 type structure has been systematically tuned from 1.0 nm to 8.5 nm with an interval of *ca.* 0.75 nm by stepwise addition of a phenyl ring into the organic linker.^16^ More precise adjustments can be reached using smaller spacers and/or changing the ligand side groups.^17,18^ Nevertheless, just like conventional hosts, the pore sizes of MOFs can be hardly tuned with a precision below 0.1 nm.

The structural transformations of flexible MOFs can be used to distinguish different guest molecules including gases.^19,20^ The gate-opening processes of flexible MOFs usually lead to selective adsorption of guest molecules with smaller sizes and higher polarities. Being similar to biomacromolecules, the adaptive dynamism of MOFs can realize molecular recognition without strict requirements of the pore sizes.^21,22^ However, on-demand control of framework flexibility is even more difficult than for pore size, because little knowledge has been developed to understand the relationship between framework structure (such as pore size) and framework flexibility.^23,24^

Besides changing the ligand spacer and/or side group, the modular structures of MOFs provide an additional parameter, *i.e.*, the metal ion, for precise adjustment of the pore size. The frequently used first-row divalent transition metal ions (with octahedral geometry) possess ionic radii gradually changing from 0.97 Å for Mn(ii) to 0.88 Å for Zn(ii), a range in which the intervals between adjacent elements are far below 0.01 nm.^25^ To fully utilize the different radii of metal ions and to realize the recognition of small gas molecules, ultramicroporous structures with extremely short bridging ligands are necessary.^26,27^ Accordingly, three isostructural ultramicroporous metal azolate frameworks, namely [Zn_3_(vtz)_6_], [Mn_3_(vtz)_6_], and [Cd_3_(vtz)_6_] (MAF-123-Zn, MAF-123-Mn, and MAF-123-Cd; and for clarity hereafter denoted as **Zn**, **Mn**, and **Cd**, respectively; Hvtz = 1,2,3-triazole)^28,29^ were selected for the study.

## Results and discussion

**Fig. 1:**
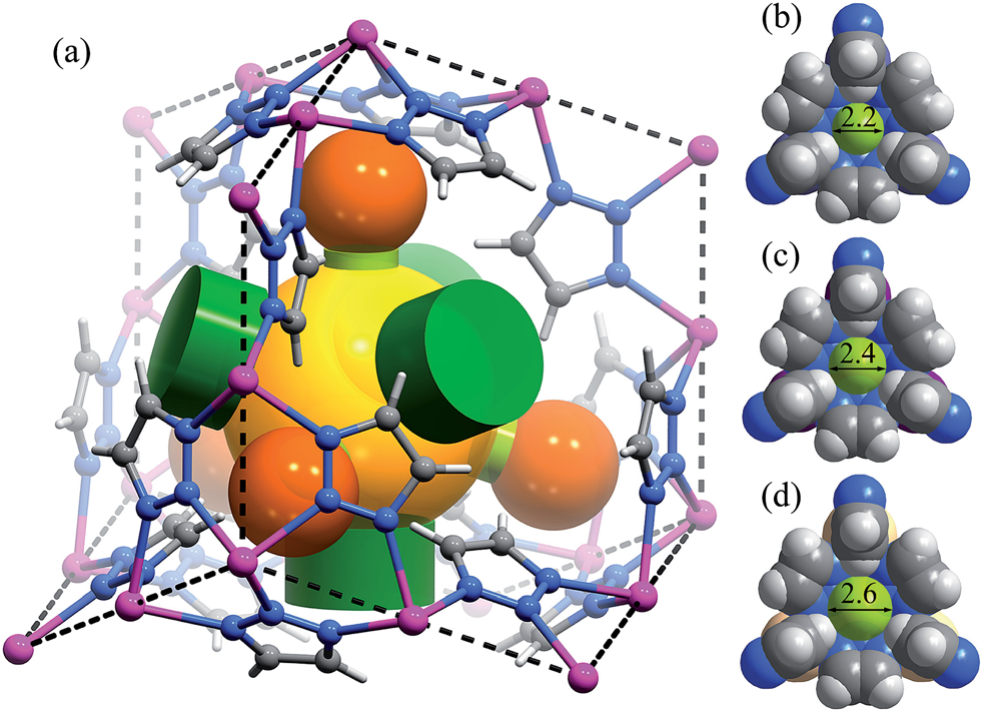
(a) The framework and pore structure of [M_3_(vtz)_6_] (black dashed lines: linkers of the **dia** topology, yellow sphere: cavity of the pore system, green cylinders: channels connecting adjacent cavities, orange spheres: pore-surface pockets, and light green cylinders: pocket entrances). (b)–(d) Structures of the pore-surface pockets of **Zn**, **Mn** and **Cd**, respectively, in a static point of view (entrances are highlighted by light-green spheres with aperture diameters in the unit of Å).

High-quality single crystals of **Zn**, **Mn**, and **Cd** were successfully obtained through high-temperature hydrothermal reactions (Fig. S1 and S2†), which enabled precise determination of the host–guest structures. [M_3_(vtz)_6_] is a three-dimensional 4-connected **dia** type coordination framework constructed from vertex sharing Kuratowski-type M_5_(vtz)_6_-tetrahedra (Table S1†),^30^ which embeds a pore system with the same **dia** topology consisting of small cavities and even smaller connecting channels (Fig. 1a). Benefiting from very short organic linkers (just one N–N bond length), the 0.01 nm differences between the metal ions effectively transfer to the pore sizes. The most important feature of the pore structure of [M_3_(vtz)_6_] is the presence of pore-surface pockets, which are defined by six vtz^–^ ligands arranged alternatively either parallel or perpendicular to the pore surface. The three parallel ones provide their electronegative N atoms for the pocket bottom, while the three perpendicular ones provide their electropositive H atoms for the pocket entrance. For **Zn**, **Mn**, and **Cd**, the entrance diameters are 2.2, 2.4, and 2.6 Å and the inner diameters/depths are 3.4/2.0, 3.6/2.1, and 3.8/2.2 Å, respectively (Fig. 1b–d).

**Fig. 2:**
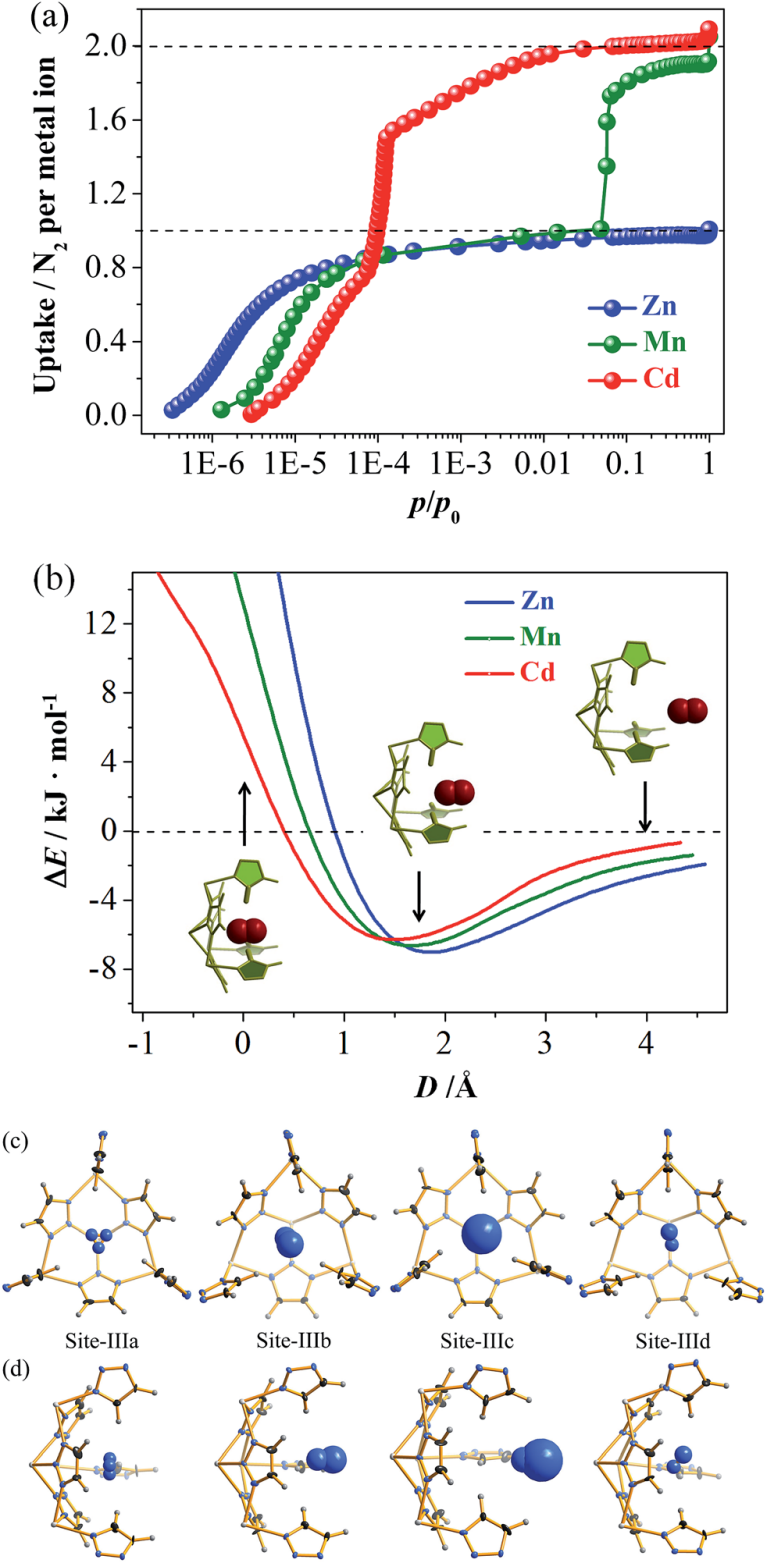
(a) Stoichiometric/non-stoichiometric N_2_ adsorption isotherms of [M_3_(vtz)_6_] measured at 77 K. (b) PES of a N_2_ molecule inserting into the pocket calculated using DFT based on rigid structures. *D* is the distance between the pocket entrance and the molecular centroid of N_2_. The insets are the three typical host–N_2_ structures for **Mn**. (c) Top and (d) side views of host–guest configurations of four kinds of pocket in the single-crystal structure of **Cd·2N_2_**. Thermal ellipsoids are drawn at 50% probability. The N_2_ molecule at Site-IIIa exhibits symmetry-induced 3-fold disorder.

N_2_ adsorption isotherms of **Zn**, **Mn**, and **Cd** show remarkably different shapes and uptakes (Fig. 2a, S3 and S4†).^28^ Specifically, **Zn** shows a typical type-I isotherm with a saturation uptake of 1.0 N_2_/Zn, while **Mn** shows a two-step isotherm with saturation uptakes of 1.0 and 1.9 N_2_/Mn. **Cd** also shows a two-step isotherm, but its saturation uptakes are 0.8 and 2.0 N_2_/Cd. The observation of stoichiometric saturation uptakes, including 1.0 N_2_/Zn, 1.0 N_2_/Mn, and 2.0 N_2_/Cd, indicates the formation of commensurate and ordered host–guest structures under the corresponding conditions. The trends of initial onset adsorption pressure and total N_2_ uptake can be roughly explained by the different pore sizes, but the experimental pore volumes cannot fit the theoretical values (Table S2 and Fig. S5†).

The N_2_ adsorption mechanisms were studied using single crystal X-ray diffraction (SCXRD) at different gas loadings, with successful measurements obtained for [Zn_3_(vtz)_6_]·3N_2_ (**Zn·N_2_**), [Cd_3_(vtz)_6_]·1.5N_2_ (**Cd·0.5N_2_**), and [Cd_3_(vtz)_6_]·6N_2_ (**Cd·2N_2_**). The host framework in **Zn·N_2_** is identical to **Zn** (Table S1†). The channel center (Site-I) and the cavity center (Site-II) are fully occupied to give a total of 3 N_2_ molecules per formula unit (hereafter, per formula unit is denoted as /unit) of **Zn**, being consistent with the experimental saturation uptake of 1.0 N_2_/Zn. The N_2_ molecule at Site-I exhibits a significantly smaller thermal parameter and less disorder (Fig. S6†). Grand Canonical Monte Carlo (GCMC) simulations further confirmed Site-I as the primary adsorption site (Fig. S7†). Although the N_2_ molecules show more disorder due to the large pore size and partial occupancy, the host–guest structure and relative binding affinities of Site-I and Site-II in **Cd·0.5N_2_** are very similar to those of **Zn·N_2_** (Fig. S8†).

Interestingly, **Cd·2N_2_** possesses a distorted host framework with a slightly expanded (0.6%) unit cell (Table S1†), giving two kinds of **dia** cage (Cage-I and Cage-II) and two kinds of channel (Site-Ia and Site-Ib), as well as four kinds of pore-surface pockets (denoted as Site-IIIa to Site-IIId, Fig. 2c, d and S9†). Summing the N_2_ molecules at Site-I and Site-III gives a total occupancy of 6 N_2_/unit, which is consistent with the experimental saturation uptake of 2 N_2_/Cd. It should be noted that the void ratio of **Cd·2N_2_** is even slightly smaller than that of **Cd** when adopting the van der Waals radius of a nitrogen atom (1.55 Å) as a probe (Fig. S5†). This fact demonstrates that the sudden increase of N_2_ uptake originates from the framework deformation rather than the host expansion (Fig. S10†). Density functional theory (DFT) simulations produced potential energy surfaces (PESs) for inserting N_2_ molecules into the rigid pockets (Fig. 2b). Outside the pocket, the host–guest binding is energetically favored and follows a **Zn** > **Mn** > **Cd** trend. Inside the pocket, the energy trends are reversed but still indicate better accessibility for larger pockets.

To further investigate the molecular recognition behaviors of the molecular pockets, H_2_, possessing a smaller molecule size (Table S3†), was selected as a guest. All of the isotherms measured at 77 K show type-I characteristics without obvious saturation, which is typical for H_2_ because this gas can interact weakly with most materials. At 1.2 atm, the H_2_ uptakes of **Zn**, **Mn** and **Cd** reach 1.13, 2.12, and 1.71 wt%, 16.6, 27.6, and 26.7 mg cm^–3^, or 1.06, 1.99, and 2.09 H_2_/M, respectively (Fig. S11†). The large H_2_ uptake of **Mn** cannot be simply explained by its small molecular weight. Instead, a more important feature of the H_2_ adsorption isotherm of **Mn** is that it has the largest slope, which is useful for practical H_2_ storage applications. For example, taking 0.1–1.2 atm as the working charge–discharge pressure range, **Mn** and **Cd** can deliver 75% and 51% of the H_2_ adsorbed at 1.2 atm, giving usable storage capacities (USCs)^31^ of 1.60 and 0.88 wt% or 20.7 and 13.6 mg cm^–3^, respectively.

**Fig. 3:**
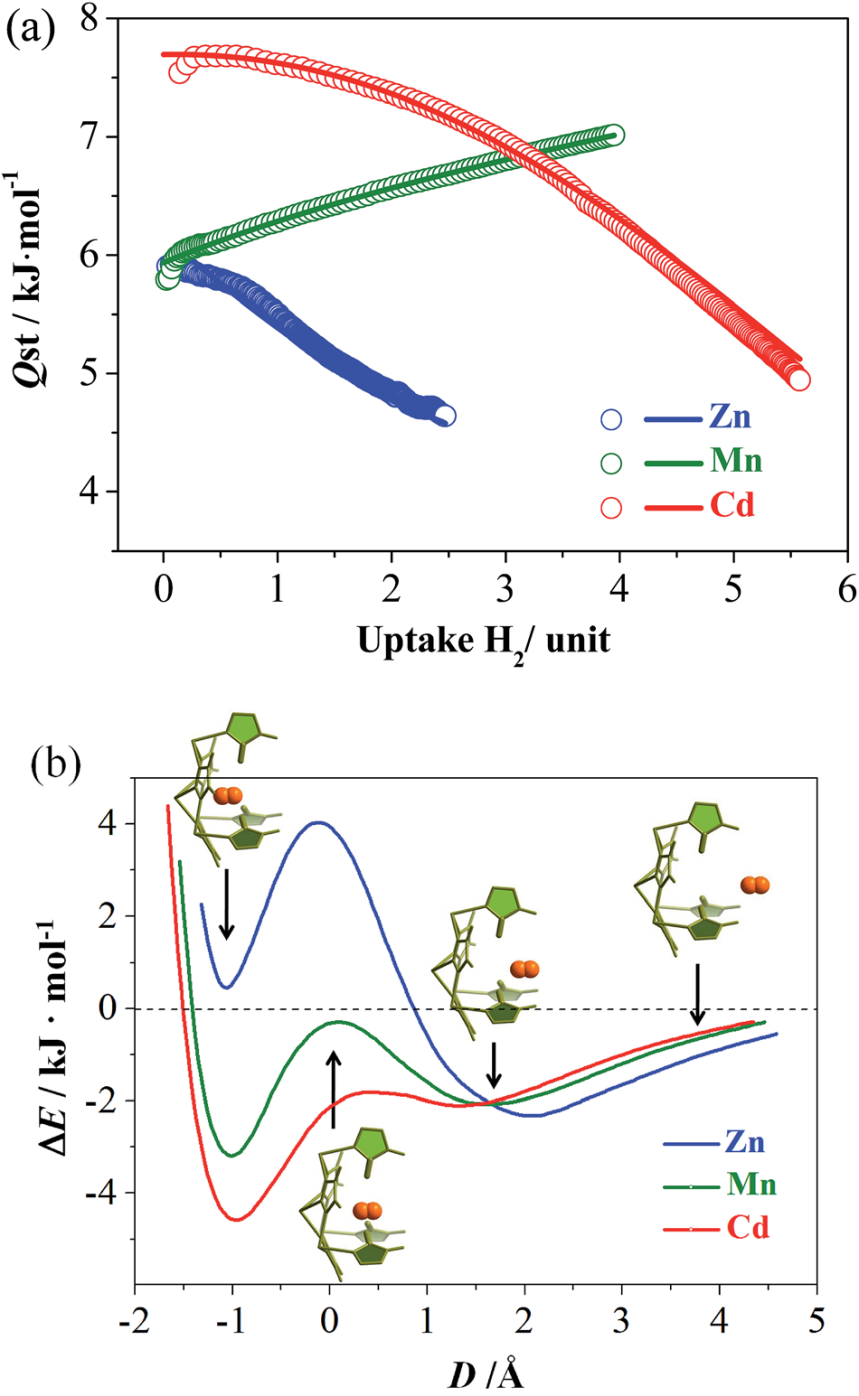
(a) The coverage-dependent H_2_ adsorption enthalpies (*Q*_st_) calculated using the Clausius–Clapeyron equation using original data without fitting (points) and using the virial equation (lines). (b) PES of a H_2_ molecule inserting into the pore-surface pockets calculated using DFT based on rigid structures. *D* is the distance between the pocket entrance and the molecular centroid of H_2_. The insets are four typical host–H_2_ structures for **Mn** placed at their corresponding PES positions.

To explain the abnormal H_2_ isotherm slope of **Mn**, loading-dependent adsorption enthalpies were calculated using the Clausius–Clapeyron equation or virial equation using isotherms measured at 77 and 87 K (Fig. 3a, S11–S13†). At near-zero loading, the H_2_ adsorption enthalpies were calculated as 5.9, 5.8, and 7.5 kJ mol^–1^ (based on the Clausius–Clapeyron equation) for **Zn**, **Mn**, and **Cd**. Note that the large-pore **Cd** possesses the largest value, in contrast with conventional observations. When the loading increases, the enthalpies of **Zn** and **Cd** gradually decrease, similar to other adsorbents. Interestingly, the enthalpy of **Mn** gradually rises to 7.0 kJ mol^–1^ at 3.95 H_2_/unit, meaning that the host can adsorb H_2_ more easily at higher loadings as reflected by its relatively large isotherm slope. Generally, the adsorption enthalpy decreases as loading increases, because the adsorbate molecules are firstly adsorbed at the strongest site and finally the weakest site. The adsorption enthalpy may sometimes increase as the loading increases because of increased adsorbate–adsorbate interaction and/or structural transformation of the adsorbent. However, both the adsorbate–adsorbate and adsorbent–adsorbate interactions are extremely weak for H_2_ molecules (reflected by its boiling point and low adsorption enthalpies), so such an increasing adsorption enthalpy profile is unprecedented for H_2_.^32,33^ As H_2_ can hardly induce a structural transformation of the adsorbent,^34,35^ and it is very difficult to determine the H_2_ position in crystal structures, we calculated the PES for inserting a H_2_ molecule into the pockets of **Zn**, **Mn**, and **Cd**, using a DFT method with rigid hosts. As shown in Fig. 3b, all three compounds showed two local minima, located outside and inside the pocket, respectively. For **Zn**, the more stable one appears outside the pocket (close to Site-II), and the less stable one has a positive binding energy. On the contrary, the more stable ones of **Mn** and **Cd** are both inside the pockets, indicating that H_2_ molecules are in favor of staying outside the pockets in **Zn**, but tend to enter the pockets in **Mn** and **Cd**. In addition, the energy barriers between the two local minima are in line with **Zn** ≫ **Mn** > **Cd** ≈ 0. All of these sequences can be explained by the sizes of the pockets (including their entrances). Only the pockets of **Mn** and **Cd** are large enough for the accommodation of a hydrogen molecule. Therefore, the H_2_ adsorption of **Zn** just occurs outside the pockets, giving a normal adsorption behavior. For **Mn**, H_2_ is firstly adsorbed in the cavities and channels, giving a low zero-loading enthalpy. However, H_2_ has a great tendency to overcome the energy barrier between the cavity and the pocket, especially at higher pressures. Therefore, more and more H_2_ molecules are absorbed in the pockets (the stronger binding sites) at higher pressures, giving the abnormal enthalpy profile and large isotherm slope. The energy barrier of **Cd** is negligible due to being largest in size, so H_2_ is adsorbed in the strongest adsorption site or inside the pockets, almost from the beginning. Thus, **Cd** displays the largest zero-loading adsorption enthalpy and a normal enthalpy trend.

**Fig. 4:**
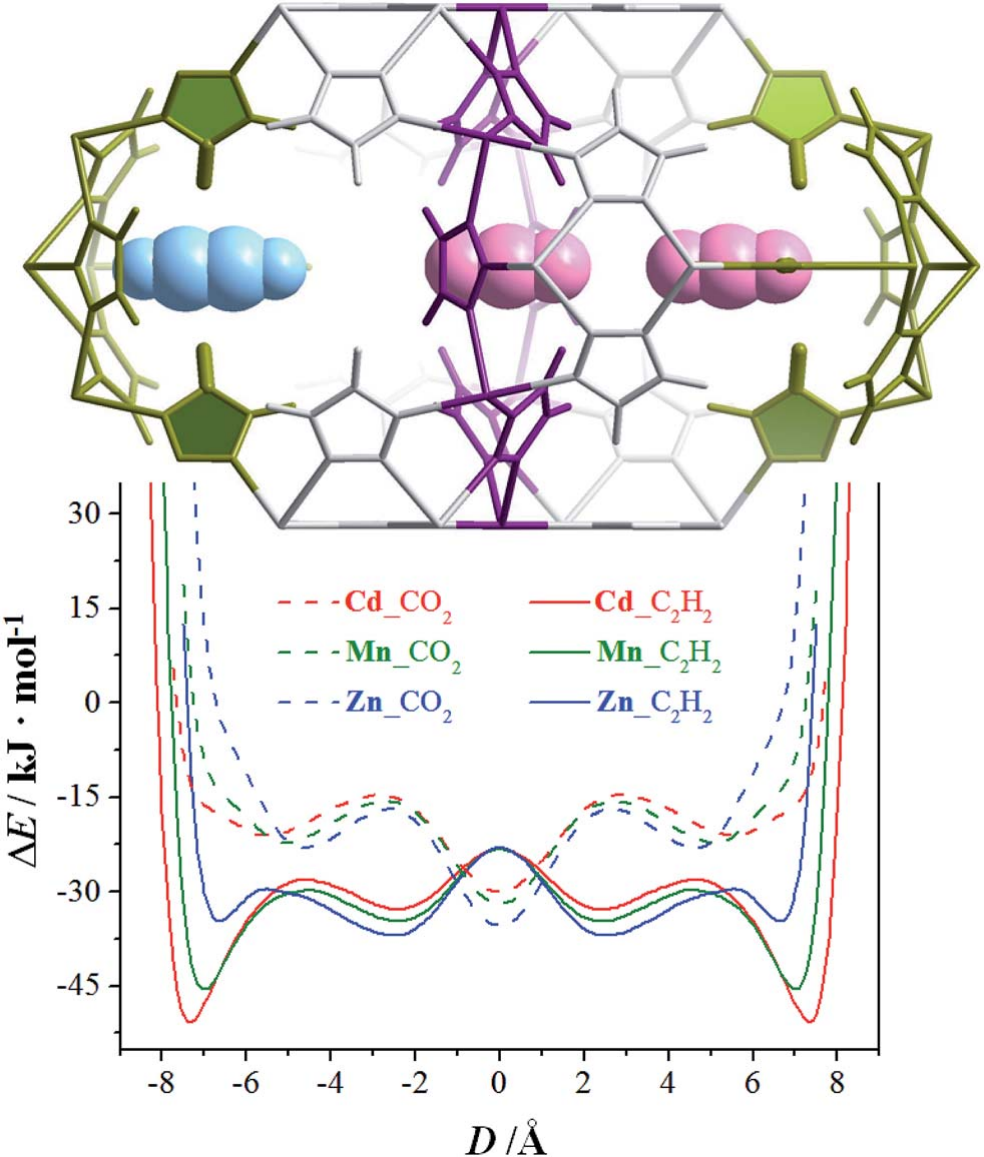
PES of a CO_2_/C_2_H_2_ molecule moving linearly between the bottoms of two pockets (connected by a channel) in [M_3_(vtz)_6_] supposing a rigid host. *D* is the distance between the centers of the channel and the gas molecule. Inset: a portion of **Cd** (scaled to fit the abscissa) with three typical guest positions.

The N_2_ and H_2_ sorption experiments demonstrated that a slight change of the metal ion size can readily control the accessibility of the pore-surface pockets. However, N_2_ and H_2_ are so small/short and can either stand or lie inside the pockets. To utilize the well-defined electrostatic fields of the pockets, we further measured adsorption isotherms for CO_2_ and C_2_H_2_ possessing large and opposite quadrupole moments, as well as larger/longer molecular sizes/shapes compared with N_2_ and H_2_ (Table S3†).^36^ At 195 K, none of the CO_2_ saturation uptakes of **Zn**, **Mn**, and **Cd** reach 4.0 CO_2_/unit or any other stoichiometric values (Fig. S14†), indicating that the guest molecules are disordered outside of the pockets. The C_2_H_2_ adsorption isotherms of **Cd** and **Mn** both exhibit one-step behavior, and their saturated uptakes are both 1.98 C_2_H_2_/M or 5.94 C_2_H_2_/unit, being close to the second-step saturated uptake of N_2_. The adsorption isotherm of **Zn** exhibits a multi-step behavior. The saturation uptakes of the two most obvious steps are 1.09 and 2.08 C_2_H_2_/Zn, the latter of which indicates that C_2_H_2_ must have entered the pockets. The PES of a CO_2_/C_2_H_2_ molecule moving linearly between the bottoms of two pockets in [M_3_(vtz)_6_] straightforwardly demonstrates that C_2_H_2_ can insert into the pockets of all three compounds, and that the pocket accessibilities are proportional to the metal radius. On the contrary, CO_2_ cannot enter any of the pockets in any of the compounds as no local minima occur for inside the pockets (Fig. 4). Obviously, [M_3_(vtz)_6_] can efficiently recognize the different quadrupole moments of CO_2_ and C_2_H_2_ to realize unprecedented adsorption selectivities. It is worth noting that they can usually choose the best orientations to adapt to the electrostatic field of the pore surface, even in ultra-microporous MOFs like CPL-2 and MAF-2 showing relatively high CO_2_/C_2_H_2_ selectivities.^31,37^

**Fig. 5:**
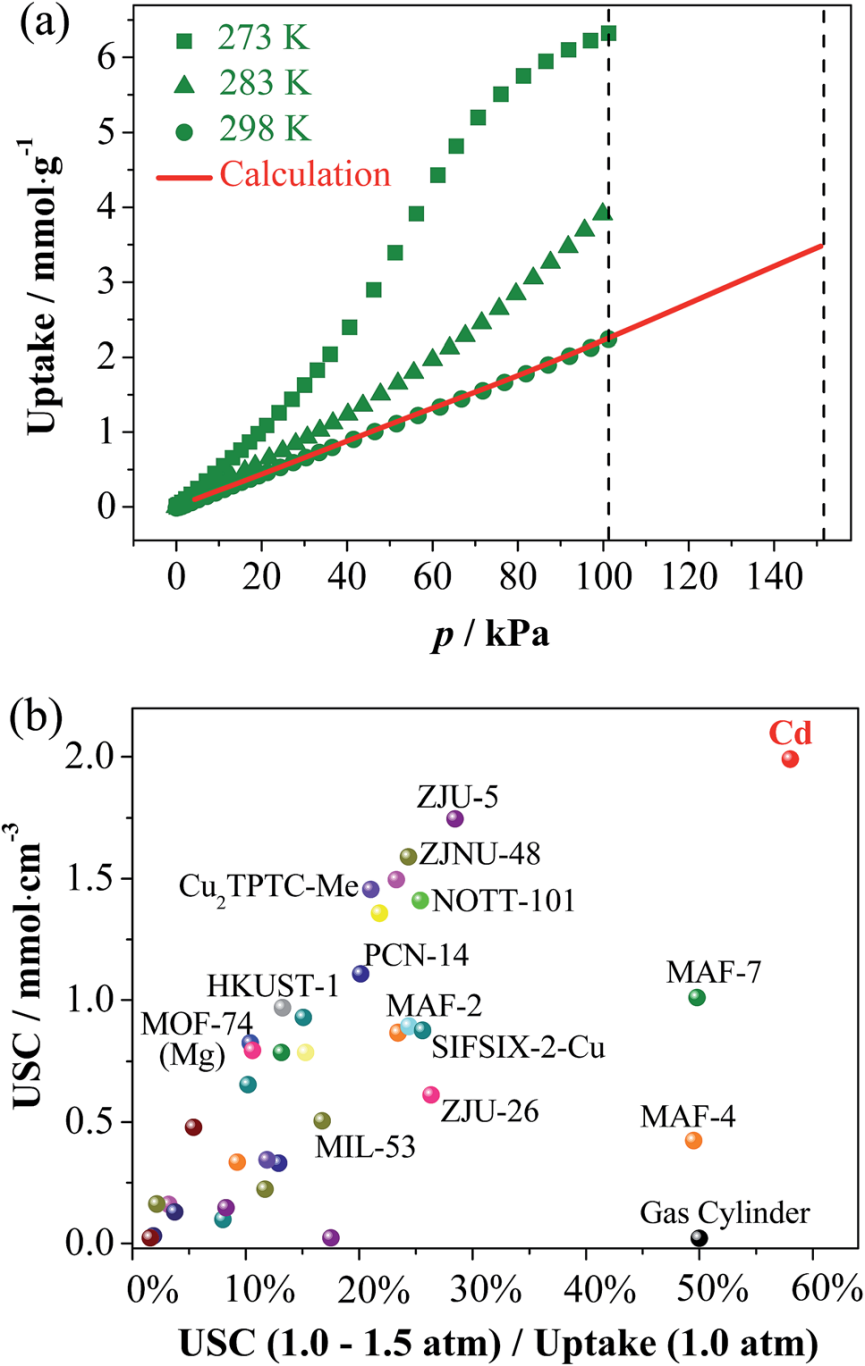
(a) C_2_H_2_ adsorption isotherms of **Cd** at 273, 283 and 298 K. The predicted isotherm was obtained based on the Clausius–Clapeyron equation and isotherms measured at 273, 283, and 298 K. The two dashed lines represent the practical working limits of the charging and discharging pressures. (b) Comparison of the USCs and utilization ratios for C_2_H_2_ storage parameters of representative MOFs.

As predicted from the low-temperature isotherms, the CO_2_ adsorption of all three compounds at ambient temperatures is poor with the uptakes no more than 2.23 mmol g^–1^ at 1 atm (Fig. S15†). The C_2_H_2_ uptakes of **Zn** and **Mn** are also quite low, indicating that their gate-opening pressures at ambient temperatures are higher than 1 atm. Interestingly, the C_2_H_2_ uptake of **Cd** at 1.0 atm is relatively low (2.23 mmol g^–1^) at 298 K but very high (6.34 mmol g^–1^) at 273 K (Fig. 5a). Also, the 273 K isotherm shows an obvious S-shape, indicating that the gate-opening pressure is lower and higher than 1 atm at 273 and 298 K, respectively. Such a large adsorption difference at two similar temperatures demonstrates a large slope in the isotherm of 298 K above 1 atm, which is very useful for obtaining a large USC between 1.0 and 1.5 atm (the practical compressed and discharged limits of pure C_2_H_2_). Since pure C_2_H_2_ explodes above 2 atm, it is stored in gas cylinders below 1.5 atm (for safety) and because gas storage systems cannot discharge below 1.0 atm (cannot outflow automatically), it can provide very limited USCs. Although some porous materials can adsorb large amounts of C_2_H_2_ at ambient temperature and pressure (Table S4†),^38^ their USCs are usually very low because saturation is almost or already reached at 1.0 atm. Theoretically, a porous material with an S-shape isotherm whose inflection point is located in the working pressure region must be beneficial in improving the USC. Obviously, a good storage material/method should have not only a high USC but also a low wasting uptake at 1.0 atm.

Based on adsorption isotherms measured at 273, 283, and 298 K, the 298 K isotherm was extrapolated to give an uptake of 3.53 mmol g^–1^ at 1.5 atm (Fig. 5a and S16†), meaning a USC (at 1.0–1.5 atm) of 1.30 mmol g^–1^ or 1.99 mmol cm^–3^ was determined, being 98 times that of a gas cylinder (0.0204 mmol cm^–3^), and also much higher than all other known adsorbents (Fig. 5b and Table S4†). Besides USC, the relative ratio between USC and the wasting uptake at 1.0 atm can also be used as a specific parameter (denoted as the C_2_H_2_ utilization ratio) to evaluate the efficiency of a storage system, which is determined by the shape of the adsorption isotherm. The C_2_H_2_ utilization ratios of porous materials are generally much lower than 50% because their type-I isotherms or quasi type-I isotherms exhibit smaller slopes at higher pressures. A few porous materials with weak C_2_H_2_ adsorption affinities can show linear isotherm shapes (such as MAF-4 (ref. 39) and MAF-7 (ref. 40)), just like the gas cylinder, to give approximately 50% utilization ratio, but their USCs are relatively low. Remarkably, the C_2_H_2_ utilization ratio of **Cd** reaches 58%, because the isotherm increases along with the pressure in the working pressure range.

## Conclusions

By using the tiny differences between metal ions, the pore structures of a series of isostructural ultramicroporous MOFs have been continuously regulated with the precision of a hundredth of a nanometer leading to interesting size and quadrupole-moment recognition behaviors, being useful for gas adsorption, separation and storage. *In situ* SCXRD analyses and computational simulations played critical roles in revealing the structural and energetic mechanisms. For instance, without SCXRD, the great structural difference between **Cd** and **Cd·2N_2_** would be ignored as in conventional cases (Fig. S1†). This work also demonstrates the possibility and strategy for achieving a reversed adsorption sequence at energetically different adsorption sites.

## Conflicts of interest

There are no conflicts to declare.
