# Supplementary material for: Hyperfine adjustment of flexible pore-surface pockets enables smart recognition of gas size and quadrupole moment
Source: Chem Sci. 2017 Sep 11;8(11):7560–5. doi: 10.1039/c7sc03067c (PMC5676252; doi:10.1039/c7sc03067c)
Supplement: Supplementary file 1 [file SC-008-C7SC03067C-s001.pdf]

Electronic Supplementary Material (ESI) for Chemical Science.
This journal is © The Royal Society of Chemistry 2017

Electronic Supplementary Information

Hyperfine Ajustment of Flexible Pore-Surface Pockets Enables Smart Recognitions of Gas Size and Quadrupole Moment

Chun-Ting He, Zi-Ming Ye, Yan-Tong Xu, Dong-Dong Zhou, Hao-Long Zhou, Da Chen, Jie-Peng Zhang* and Xiao-Ming Chen

MOE Key Laboratory of Bioinorganic and Synthetic Chemistry, School of Chemistry, Sun Yat-Sen University, Guangzhou 510275, China

*E-mail: zhangjp7@mail.sysu.edu.cn

Supplementary Index

1. **Experimental Section**
2. **Table S1** Crystallographic data and structure refinement details.
3. **Table S2** Comparison of the pore volumes calculated from the experimental N_2 uptakes and from crystal structures.
4. **Table S3.** Physical parameters of H_2, N_2, CO_2, and C_2H_2.
5. **Table S4** Summary of C_2H_2 storage performances in representative MOFs.
6. **Fig. S1** PXRD patterns of [M_3(vtz)$_6$] and [Cd_3(vtz)$_6$]·6N_2.
7. **Fig. S2** Thermogravimetry curves of [M_3(vtz)$_6$].
8. **Fig. S3.** N_2 adsorption isotherms for [M_3(vtz)$_6$] at 77 K and 87 K.
9. **Fig. S4.** Loading dependent N_2 adsorption enthalpies of [M_3(vtz)$_6$].
10. **Fig. S5** Probe-dependent crystallographic void ratios of [M_3(vtz)$_6$].
11. **Fig. S6** SCXRD structure of **Zn·N$_2$**.
12. **Fig. S7** A representative GCMC-derived density field of the N_2 locations in [M_3(vtz)$_6$] under very low N_2 pressure.
13. **Fig. S8** SCXRD structure of **Cd·0.5N$_2$**.
14. **Fig. S9** Host-guest configurations observed in the single-crystal structure of **Cd·2N$_2$**.
15. **Fig. S10** Comparison of the experimental and simulated N_2, CO_2, and C_2H_2 saturation uptakes in [M_3(vtz)$_6$].
16. **Fig. S11** H_2 adsorption isotherms for [M_3(vtz)$_6$] at 77 K.
17. **Fig. S12** H_2 adsorption isotherms for [M_3(vtz)$_6$] at 87 K.
18. **Fig. S13** Virial fitting of H_2 adsorption isotherms.
19. **Fig. S14** CO_2 and C_2H_2 sorption isotherms measured at 195 K.
20. **Fig. S15** CO_2/C_2H_2 adsorption isotherms of [M_3(vtz)$_6$] at 273 K and 298 K.
21. **Fig. S16** Loading-dependent C_2H_2 adsorption enthalpy derived from isotherms measured at 273 and 283 K.
22. **Supplementary References**

Experimental Section

Materials and Physical Measurements. All reagents were commercially available and used as received without further purification. Powder X-ray diffraction (PXRD) data were recorded on a Bruker D8 Advance diffractometer (Cu Kα) at room temperature. Thermogravimetry analyses were performed at a rate of 5 °C/min under N_2 using a TA Q50 system. Gas sorption isotherms for N_2, H_2, CO_2 and C_2H_2 were measured with an automatic volumetric sorption apparatus Micromertics ASAP 2020M. Before each sorption experiment, the sample was treated by MeOH to exchange its guests and heated at 100 °C under high vacuum for 5 h to remove the remnant solvent molecules. The temperatures were controlled by a liquid-nitrogen bath (77 K), liquid-argon bath (87 K), acetone−dry ice bath (195 K), or a water bath (273, 283, and 298 K). Because we cannot measure the isotherm of C_2H_2 to 1.5 atm, the 298 K isotherm at 1.0-1.5 atm need to be predicted. On the other hand, the S-shaped isotherm just with its low pressure section is unable to be predicted by any known isotherm models, so we used two isotherms measured at 273 and 283 K to calculate the loading-dependent adsorption enthalpy profile using the Clausius–Clapeyron equation, which was then combined with the 273 K isotherm to predict the 298 K isotherm using the Clausius–Clapeyron equation. As shown in Fig. 5a, the 0.0-1.0 atm section of the predicted isotherm fits well with the experimental 298 K isotherm, indicating the reliability of the 1.0-1.5 atm section of the predicted isotherm.

X-ray single-crystal structure analyses. Diffraction data were collected using a Bruker Apex CCD area-detector diffractometer (Mo-Kα). Absorption corrections were applied by using multi-scan program SADABS. The structures were solved by the direct method and refined with the full-matrix least-squares method on F^2 by the SHELXTL crystallographic software package.

Hydrogen atoms were placed geometrically. Anisotropic thermal parameters were used for all non-hydrogen atoms except the guest molecules. Crystal data and details of data collection and refinements of the compounds are summarized in Table S1. CCDC 1561837-1561845 contain the supplementary crystallographic data for this paper. These data can be obtained free of charge from The Cambridge Crystallographic Data Centre via www.ccdc.cam.ac.uk/data_request/cif.

Preparation of $[Zn_3(vtz)_6]$ (MAF-123-Zn): A mixture of Hvtz (0.035 g, 0.5 mmol), $Zn(NO_3)_2 \cdot 6H_2O$ (0.075 g, 0.25 mmol) and isopropanol (5.0 mL) was sealed in a Pyrex glass tube and kept at 210°C for 3 days, and then cooled to ambient temperature at a rate of 10 $^oC \cdot h^{-1}$ to form colorless block crystals (75% yield based on Zn). The resulted samples were washed by MeOH for several times and then heated to 100 ^oC under N_2 to finally obtain activated $[Zn_3(vtz)_6]$.

Preparation of $[Mn_3(vtz)_6]$ (MAF-123-Mn): A mixture of Hvtz (0.035 g, 0.5 mmol), $MnCl_2$ (0.032 g, 0.25 mmol) and isopropanol (5.0 mL) was sealed in a Pyrex glass tube and kept at 210°C for 3 days, and then cooled to ambient temperature at a rate of 10 $^oC \cdot h^{-1}$ to form white powder or colorless block crystals (45% yield based on Mn). The resulted samples were washed by MeOH for several times and then heated to 100 ^oC under N_2 to finally obtain activated $[Mn_3(vtz)_6]$.

Preparation of $[Cd_3(vtz)_6]$ (MAF-123-Cd): A mixture of Hvtz (0.035 g, 0.5 mmol), $CdCl_2$ (0.046 g, 0.25 mmol) and isopropanol (5.0 mL) was sealed in a in a Pyrex glass tube and kept at 210°C for 3 days, and then cooled to ambient temperature at a rate of 10 $^oC \cdot h^{-1}$ to form colorless block crystals (58% yield based on Cd). The resulted samples were washed by MeOH for several times and then heated to 100 ^oC under N_2 to finally obtain activated $[Cd_3(vtz)_6]$.

Computational calculations. All the GCMC simulations/DFT calculations were performed in the Materials Studio 5.5 package. The preferred adsorption sites and the saturation uptakes of N_2 (T = 77 K) and CO_2/C_2H_2 (T = 195 K) were simulated through the Fixed loading and Fixed pressure task in the Sorption module, respectively. The MOF framework and the gas molecule were both regarded as rigid. The simulation box consisted of four unit cell and the Metropolis method based on the Universal forcefield (UFF) was used. Mulliken charges calculated from DFT were all adopted for all the host frameworks and gas molecules. The density fields of all the gas adsorption were generated from the GCMC simulations with the grid interval of 0.4 Å. The cutoff radius was chosen as 15.5 Å for the Lennard-Jones potential, and all the equilibration steps and production steps were both set as 5×10^6.

The geometry optimizations and binding energies were calculated by the PDFT method through the Dmol^3 module. The widely used generalized gradient approximation (GGA) with the Perdew-Burke-Ernzerhof (PBE) functional and the double numerical plus d-functions (DND) basis set were used for the non-metal atoms. An accurate effective core potentials (ECP) was employed for the metal atoms. Considering that a real description of the adsorption of framework with gas molecule can only be obtained from an appropriate balance of all interactions. Thus, the DFT including the long-range dispersion correction (DFT-D) was also taken into account using the Grimme (G06) semiempirical method to describe the long-range van der Waals interactions. For all the DFT-D calculations, the energy, gradient and displacement convergence criterions were set as 1×10^{-5} Ha, 2×10^{-3} Å and 5×10^{-3} Å, respectively.

In order to further understand the accurate potential energy surface (especially for the smaller gas molecules H_2) when the guest molecular going through the pocket, we further performed DFT

calculations with model clusters using Gaussian03. The fragments or clusters were taken from the periodic structure based on the crystal data. Due to considering the chemical environment in the real systems and keeping the clusters electric neutrality, as well as cutting down the calculation time, we saturated the cleaved clusters with light atom Li and H. Moreover, we fixed the coordination bond in the pocket (highlighted in red line) because of keeping the slight difference among the three type of coordination bond after optimization. So a partial geometry optimization was carried out for the cluster imposing C_{3v} symmetry with B3LYP functional. We used the LANL2DZ basis set and effective core potential (ECP) for transition metal elements (Zn, Mn, Cd) and 6-31G (d) for the other atoms. The guest molecule was also optimized use B3LYP with 6-31G (d). Then we let the guest molecule go into the pocket step by step along the C_3 axis, and calculated the single point energy in every step. The binding energy was then derived as: $E = E_{(\mathrm{clusters + guest})} - E_{(\mathrm{clusters})} - E_{(\mathrm{guest})}$. Where $E_{(\mathrm{clusters +guest})}$ is the total energy of the complex combined with clusters and guest, while $E_{(\mathrm{clusters})}$ and $E_{(\mathrm{guest})}$ denoted the total energies of the single clusters and guest. As B3LYP is unsuitable to estimate the weak interactions, so we used PBEPBE density functional (adopting the Perdew, Burke, and Ernzerhof functional for both exchange and correlation, which had been thought to give much better results for weak interactions) in the energy calculation. Besides, the 6-311G (d, p) basis set are used for C, H, N, Li and LANL2DZ basis set with ECP for transition metal elements without correcting basis set superposition error effects (BSSE).

Table S1. Crystallographic data and structure refinement details

Complex	$[Zn_3(vtz)_6]$_as synthesized	$[Zn_3(vtz)_6]$_Gfree	$[Zn_3(vtz)_6]\cdot 3N_2$	$[Mn_3(vtz)_6]$_as synthesized	$[Mn_3(vtz)_6]$_Gfree
Formula	$C_4H_4N_6O_{1.33}Zn$	$C_4H_4N_6Zn$	$C_4H_4N_8Zn$	$C_4H_4N_6O_{1.01}Mn$	$C_4H_4N_6Mn$
Formula weight	222.84	201.52	229.52	207.29	191.07
Temperature (K)	93(2)	103(2)	103(2)	153(2)	103(2)
Crystal system	Cubic	Cubic	Cubic	Cubic	Cubic
Space group	*Fd*-3*m*	*Fd*-3*m*	*Fd*-3*m*	*Fd*-3*m*	*Fd*-3*m*
a/Å	17.715(3)	17.6639(19)	17.656(2)	18.143(4)	18.148(2)
V/Å^3	5559.4(15)	5511.4(10)	5503.5(12)	5972(2)	5976.8(12)
Z	24	24	24	24	24
D_c /g cm^{-3}	1.597	1.457	1.662	1.382	1.274
reflns coll.	5819	8464	5669	7129	9254
unique reflns	302	297	297	321	320
R_{int}	0.0569	0.0457	0.0401	0.0847	0.0502
R_1 [$I > 2\sigma(I)$][a]	0.0295	0.0340	0.0198	0.0293	0.0253
wR_2 [$I > 2\sigma(I)$][b]	0.0685	0.0989	0.0483	0.0748	0.0620
R_1 (all data)	0.0339	0.0354	0.0233	0.0389	0.0272
wR_2 (all data)	0.0716	0.1006	0.0503	0.0800	0.0629
GOF	1.034	1.019	1.049	1.016	1.025

Table S1. Crystallographic data and structure refinement details (continued)

Complex	$[Cd_3(vtz)_6]$_as synthesized	$[Cd_3(vtz)_6]$_Gfree	$[Cd_3(vtz)_6]\cdot 1.5N_2$	$[Cd_3(vtz)_6]\cdot 6N_2$
Formula	$C_4H_{4.08}N_6O_{0.81}Cd$	$C_4H_4N_6Cd$	$C_4H_4N_7Cd$	$C_4H_4N_{10}Cd$
Formula weight	261.64	248.54	262.54	304.61
Temperature (K)	103(2)	103(2)	103(2)	103(2)
Crystal system	Cubic	Cubic	Cubic	Cubic
Space group	*Fd*-3*m*	*Fd*-3*m*	*Fd*-3*m*	*P*2(1)3
a/Å	18.6158(19)	18.6249(18)	18.6035(13)	18.6598(7)
V/Å^3	6451.3(11)	6460.7(11)	6438.5(8)	6497.1(4)
Z	24	24	24	24
D_c /g cm^{-3}	1.617	1.533	1.625	1.868
reflns coll.	3283	9928	11971	25437
unique reflns	343	342	343	4236
R_{int}	0.0330	0.0432	0.0346	0.0372
R_1 $[I > 2\sigma(I)]$[a]	0.0246	0.0193	0.0166	0.0246
wR_2 $[I > 2\sigma(I)]$[b]	0.0678	0.0466	0.0461	0.0596
R_1 (all data)	0.0285	0.0216	0.0184	0.0255
wR_2 (all data)	0.0725	0.0484	0.0485	0.0603
GOF	1.087	1.037	1.044	1.017

$^aR_1=\Sigma||F_o| - |F_c||/\Sigma|F_o|$. $^bwR_2 =[\Sigma w(F_o^2 - F_c^2)^2/\Sigma w(F_o^2)^2]^{1/2}$

Table S2. Comparison of the pore volumes calculated from the experimental N_2 uptakes and those empirically calculated from the crystallographic void ratio and crystal density.

	**Zn**	**Mn**	**Cd**
Crystal density / g cm^{-3}	1.457	1.274	1.533
Crystallography void ratio in this work / %	34.3	41.0	44.2
Crystallography void ratio in the references / %	25.3	40.5	48.0
Crystallography pore volume in this work / cm^3 g^{-1}	0.235	0.322	0.288
Crystallography pore volume in the references / cm^3 g^{-1}	0.174	0.318	0.313
Pore volume from N_2 uptake in this work / cm^3 g^{-1}	0.173	0.371	0.298
Pore volume from N_2 uptake in the references / cm^3 g^{-1}	0.17	0.35	NA

NR = not available.

Table S3. Physical parameters of H_2, N_2, CO_2, and C_2H_2.

Gas	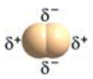	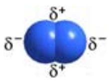	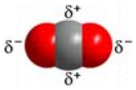	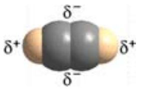
Quadrupole moment / 10^{-40} C·m^2	+2.1	-4.90	-14.9	+25.1
Polarizability / $Å^3$	0.80	1.74	2.91	3.33
Kinetic diameter / Å	2.89	3.64	3.3	3.3
van der Waals diameter / Å	2.4	3.1	3.4	3.4
van der Waals length / Å	3.15	4.2	5.4	5.8

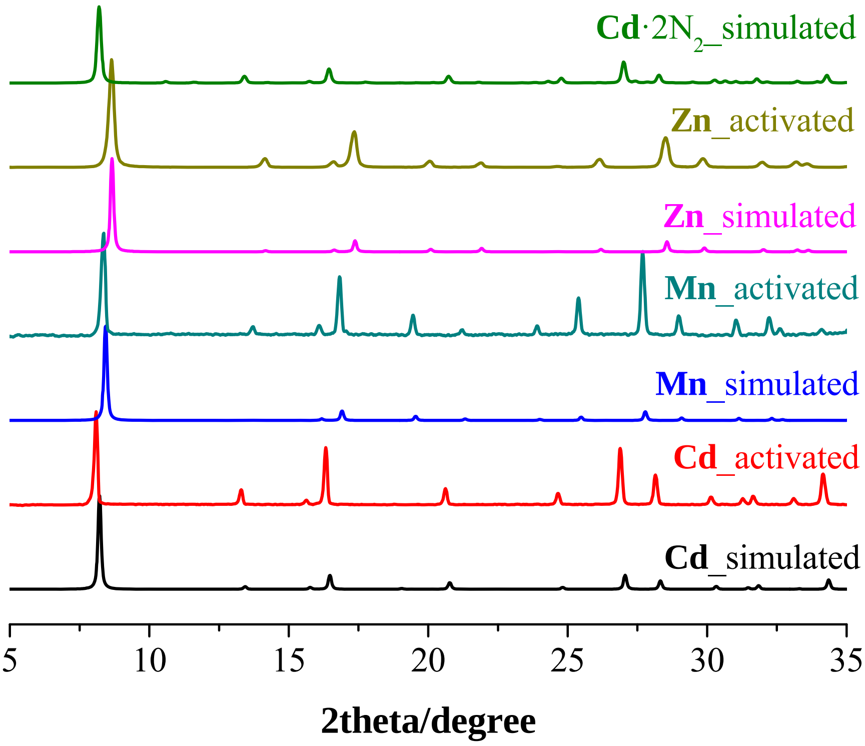

Fig. S1 PXRD patterns of $[M_3(vtz)_6]$ and $[Cd_3(vtz)_6]\cdot 6N_2$.

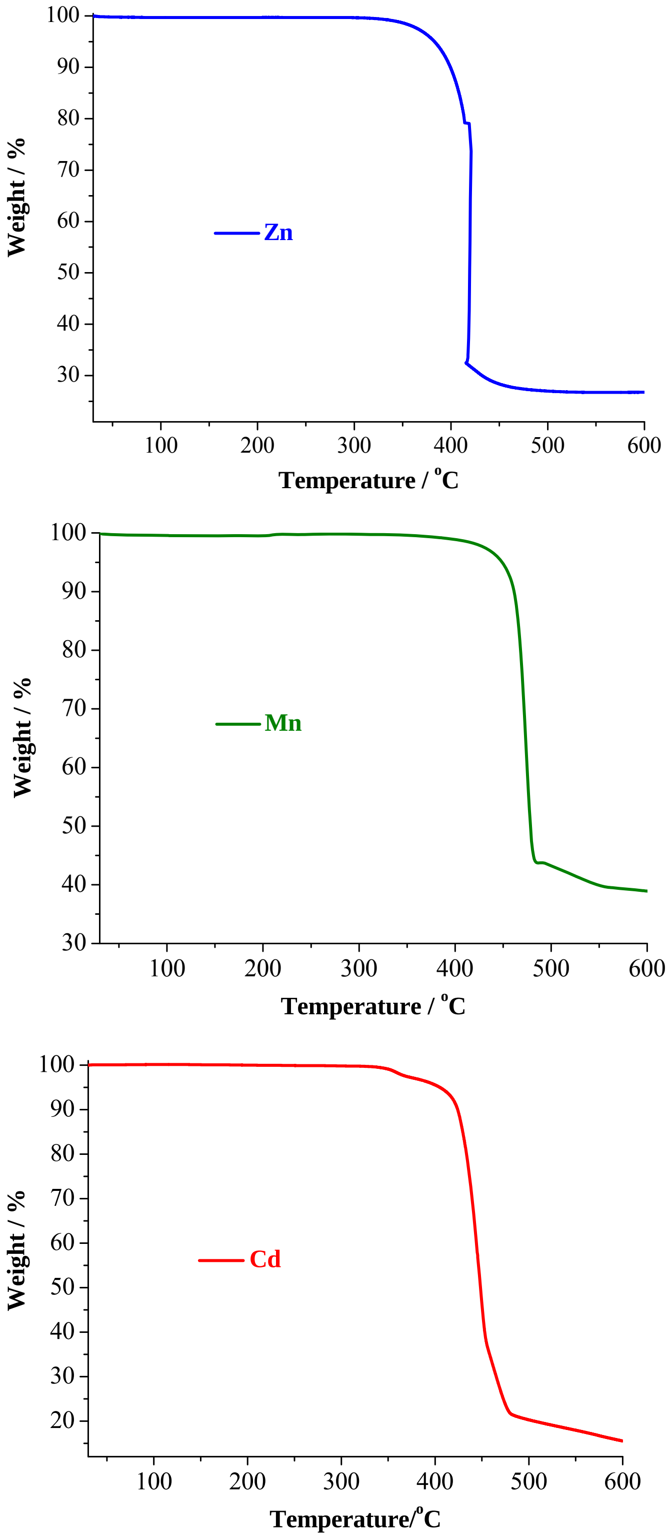

Fig. S2 Thermogravimetry curves of $[M_3(vtz)_6]$.

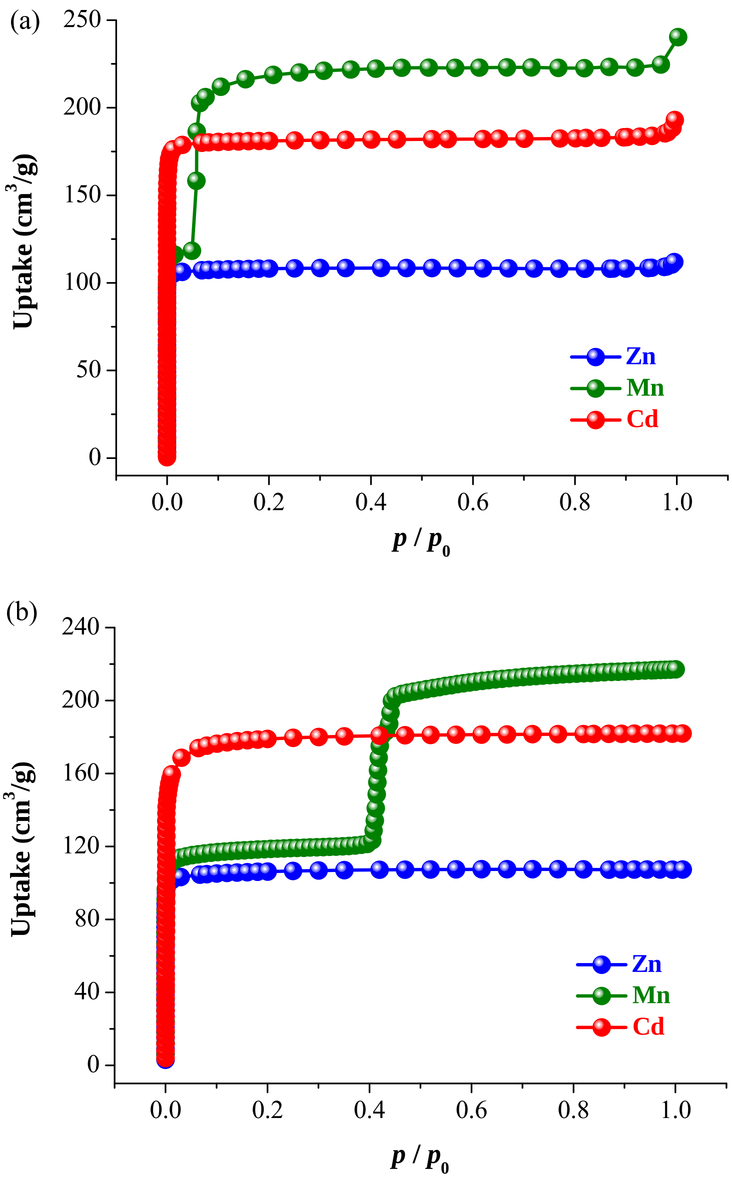

Fig. S3. N_2 adsorption (representation in volumetric capacities) isotherms for $[M_3(vtz)_6]$ at (a) 77 K and (b) 87 K.

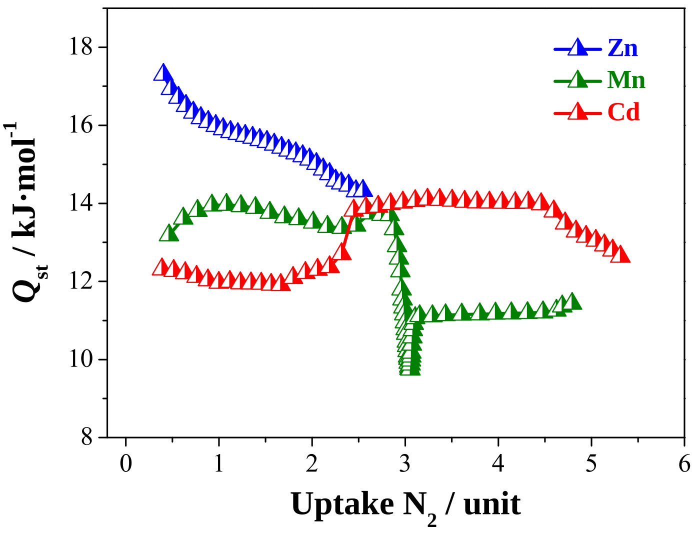

Fig. S4. Loading dependent N_2 adsorption enthalpies of $[M_3(vtz)_6]$. At the lower loading region, the N_2 adsorption enthalpies follow the sequence of **Zn** > **Mn** > **Cd**, being consistent with the expectation that smaller pore size gives stronger adsorption. When the uptakes increase, the Q_{st} of **Zn** decreases gradually due to its smallest pore size/volume. For **Mn**, the adsorption enthalpy drastically drops at ca. 3 N_2/unit, being result from the energy consumption for severe framework distortion to accommodate more N_2 molecules into the pore-surface pocket. In contrast, the Q_{st} of **Cd** exhibits an increase at ca. 2.5 N_2/unit, because its relatively large pore size allows easier framework distortion and less energy consumption.

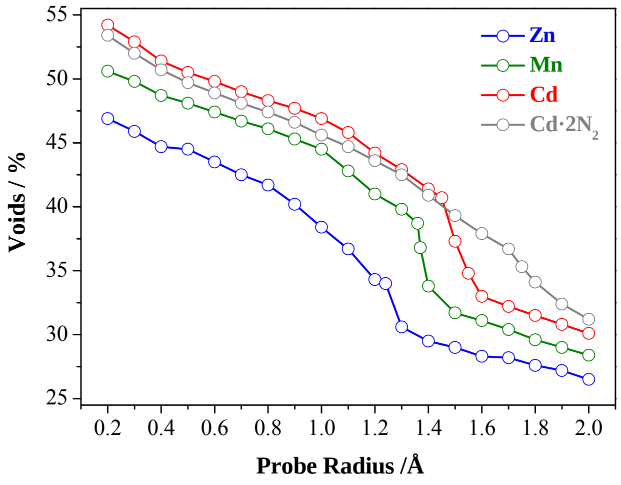

Fig. S5 Probe-dependent crystallographic void ratios of $[M_3(vtz)_6]$. The crystallographic void (solvent accessible volume) ratio is an important parameter serving as a reference for characterizing a MOF material. The void ratios were calculated by Platon using different probe radii, in which the C–H bond lengths of the structures were set as the ideal value of 1.1 Å. Using the default parameters (probe radius = 1.2 Å), their void ratios were calculated to be 34.3%, 41.0%, and 44.2%, being somewhat different with the reported values of 25.3%, 40.5%, and 48%, respectively (probe radius not reported). It can be seen that, all void ratios decrease as the probe radius increases as expected, but there are obvious jumping for **Zn**, **Mn**, and **Cd** at probe radius of *ca*. 1.3, 1.4, and 1.5 Å, respectively. This observation illustrates the presences of small pore sizes and/or of highly irregular pore surfaces being modulated in the 0.1 Å size intervals.

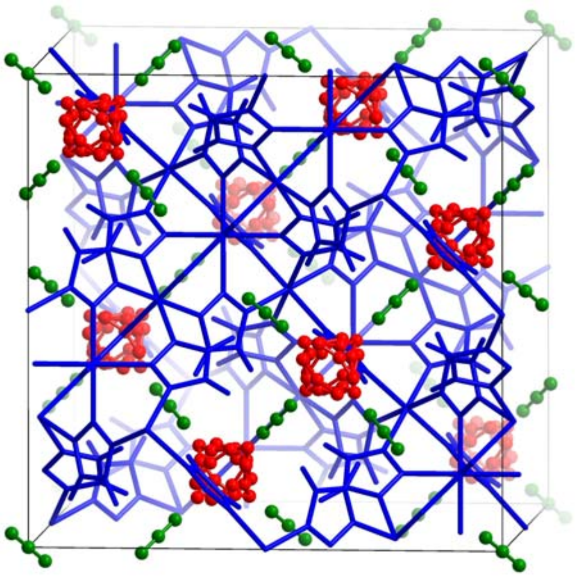

Fig. S6 SCXRD structure of **Zn·N$_2$**. The host framework is drawn as blue sticks. The N_2 molecules are drawn in the ball-and-stick mode with each stick representing a possible location of the gas molecule (Site-I: green, Site-II red).

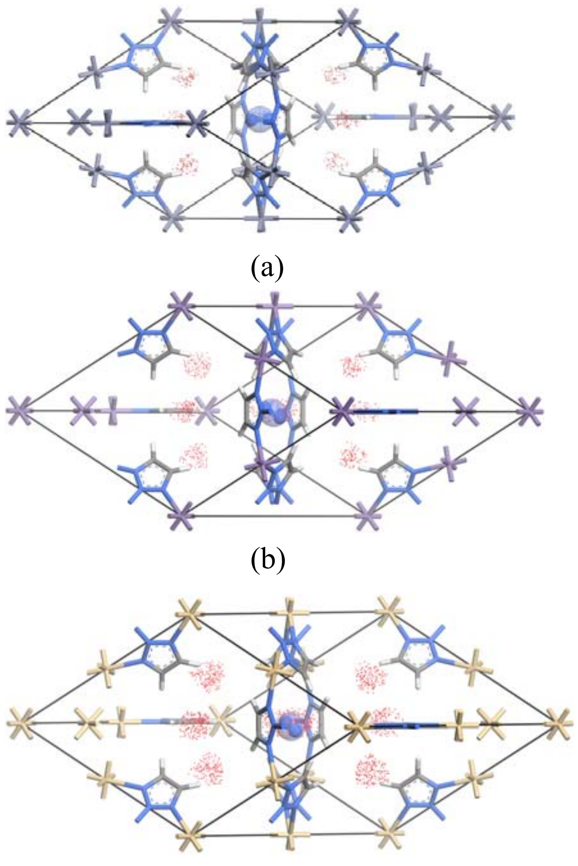

(a)

(b)

(c)

Fig. S7 A representative GCMC-derived density field of the N_2 locations in (a) **Zn**, (b) **Mn** and (c) **Cd** under very low N_2 pressure (for clarity, the reduced cell of the crystal was adopted). Note that N_2 molecules are first adsorbed inside the channels in not only **Mn** but also **Zn** and **Cd**.

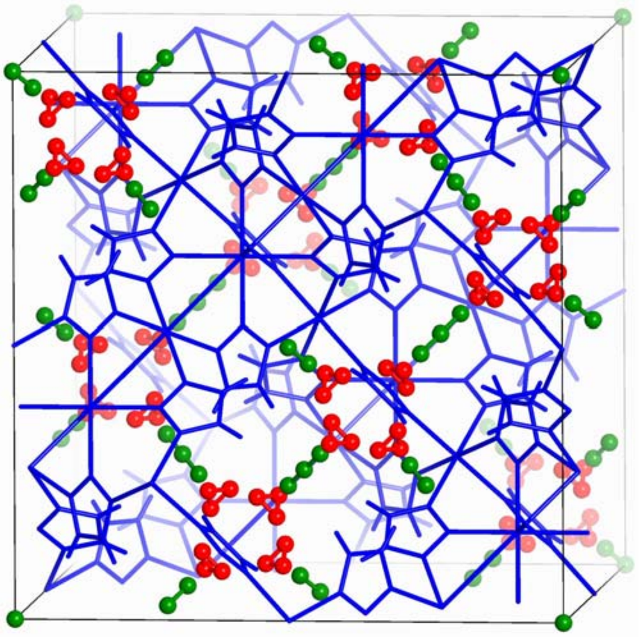

Fig. S8 SCXRD structure of **Cd·0.5N$_2$**. The host framework is drawn as blue sticks. The N_2 molecules are drawn in the ball-and-stick mode with each stick representing a possible location of the gas molecule (Site-I: green, Site-II red).

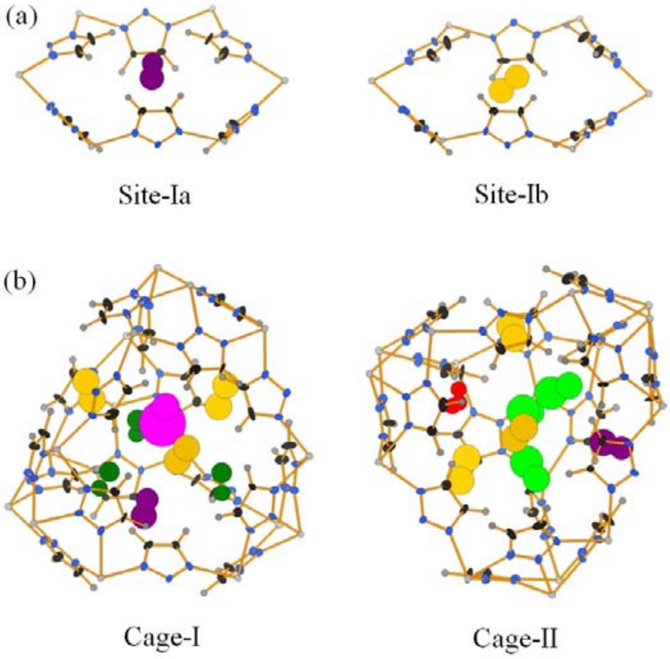

Fig. S9 Host-guest configurations observed in the single-crystal structure of **Cd·2N$_2$**. a) Local structures focused on the two kinds of channels and b) extended structures focused on the two kinds of cages. Thermal ellipsoids are drawn at 50% probability. The six crystallographically independent N_2 molecules are highlighted in six different colors.

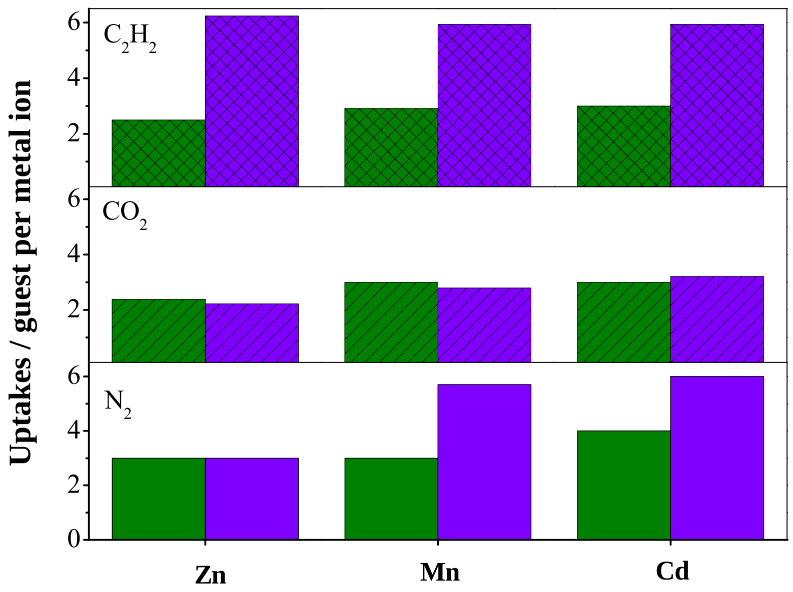

Fig. S10 Comparison of the (violet) experimental and (green) simulated (GCMC and rigid host) N_2, CO_2, and C_2H_2 saturation uptakes in [$M_3(vtz)_6$].

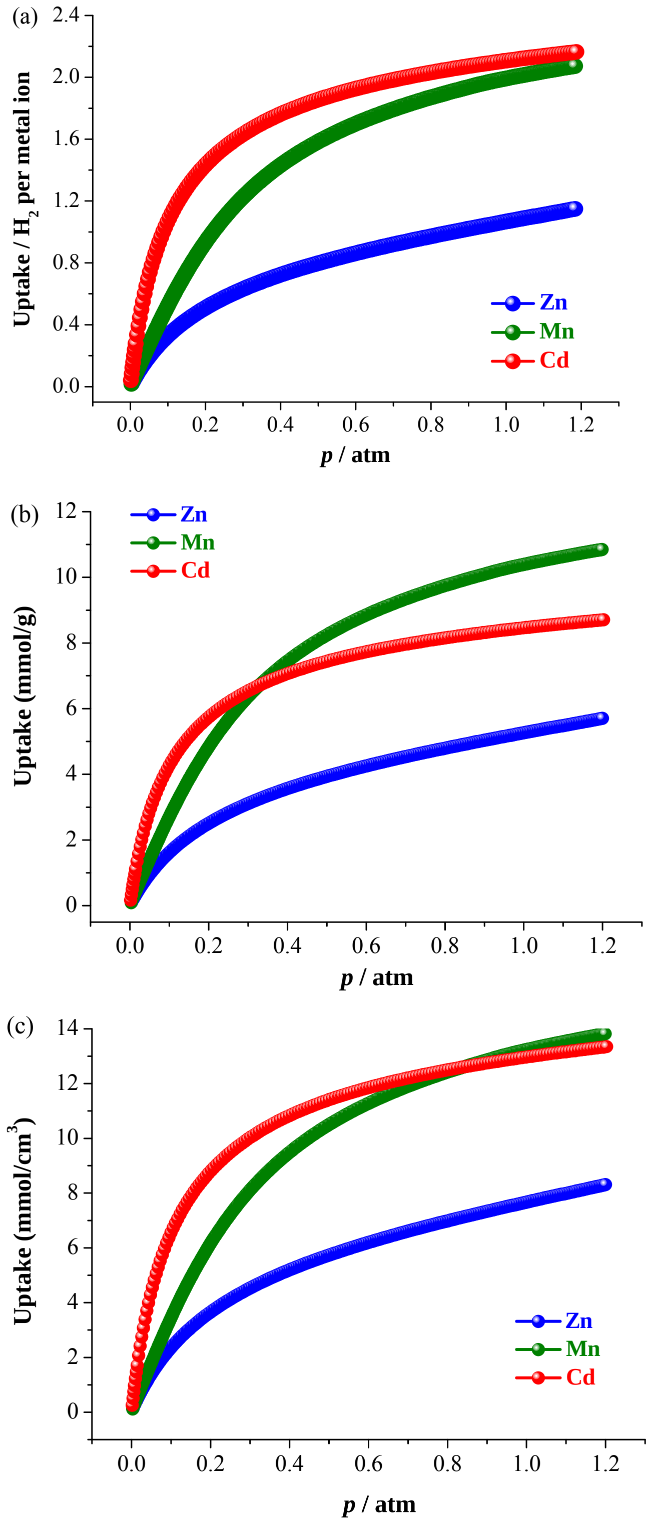

Fig. S11 H_2 adsorption isotherms for $[M_3(vtz)_6]$ at 77 K presented in the (a) molar, (b) gravimetric, and (c) volumetric values.

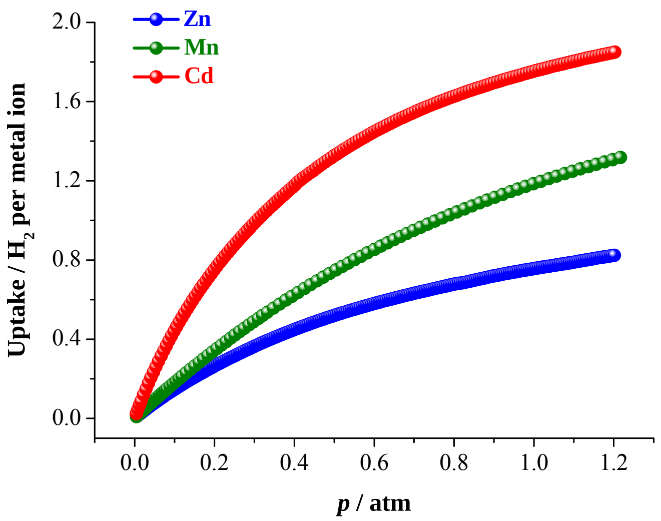

Fig. S12 H_2 adsorption isotherms for $[M_3(vtz)_6]$ at 87 K.

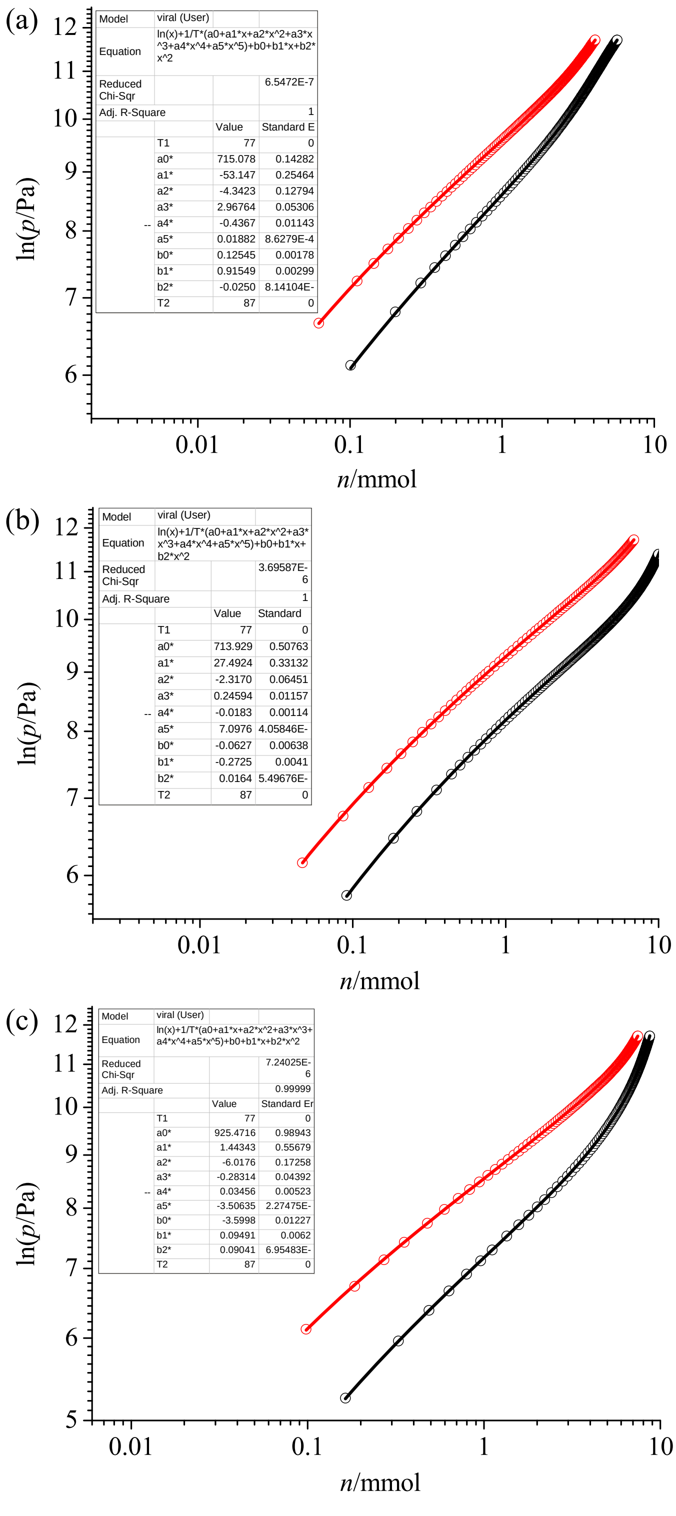

Fig. S13 Virial fitting (lines) of H_2 adsorption isotherms (points) measured at 77 K (black) and 87 K (red) for (a) [$Zn_3(vtz)_6$], (b) [$Mn_3(vtz)_6$], and (c) [$Cd_3(vtz)_6$].

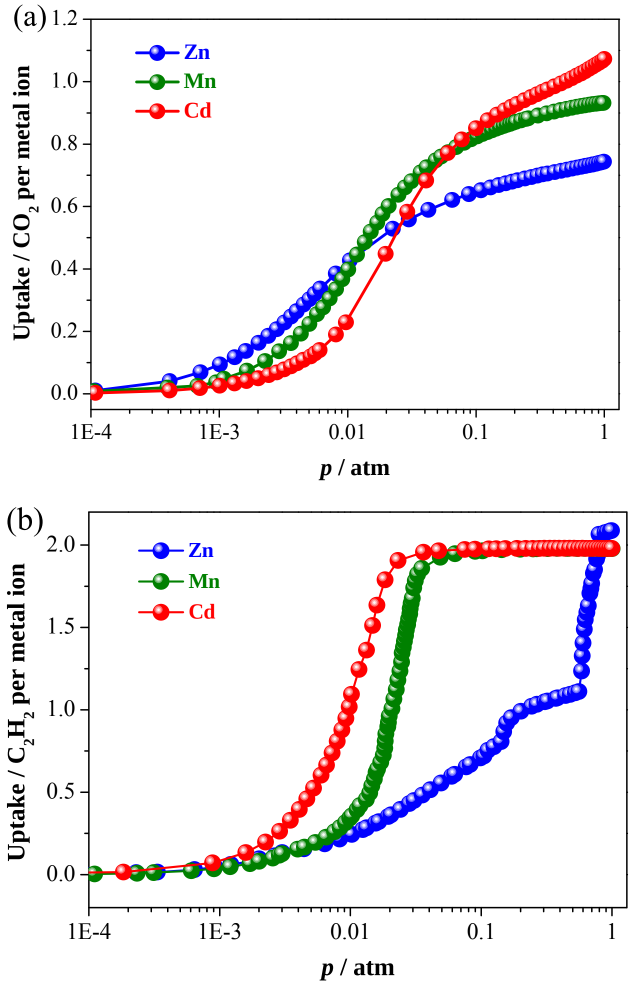

Fig. S14 (a) CO_2 and (b) C_2H_2 sorption isotherms measured at 195 K.

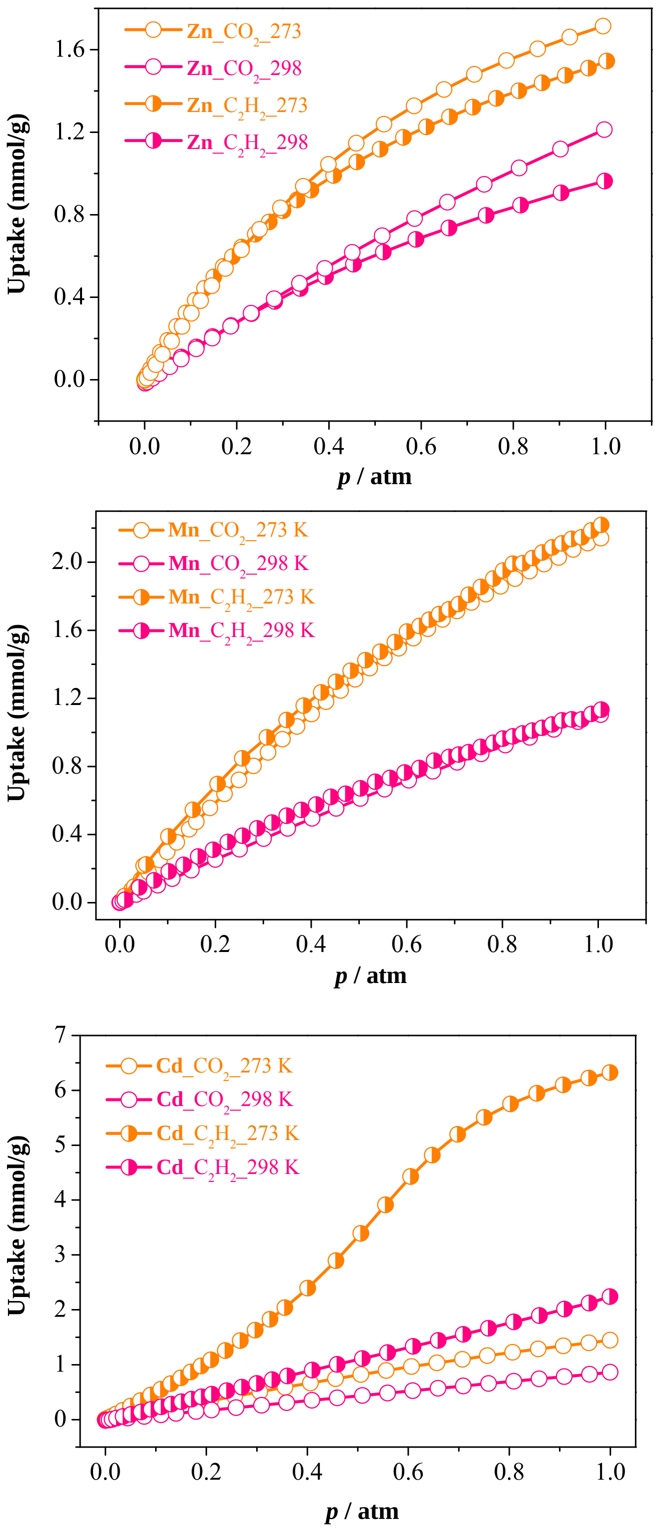

Fig. S15 CO_2/C_2H_2 adsorption isotherms of [M_3(vtz)$_6$] at 273 K and 298 K.

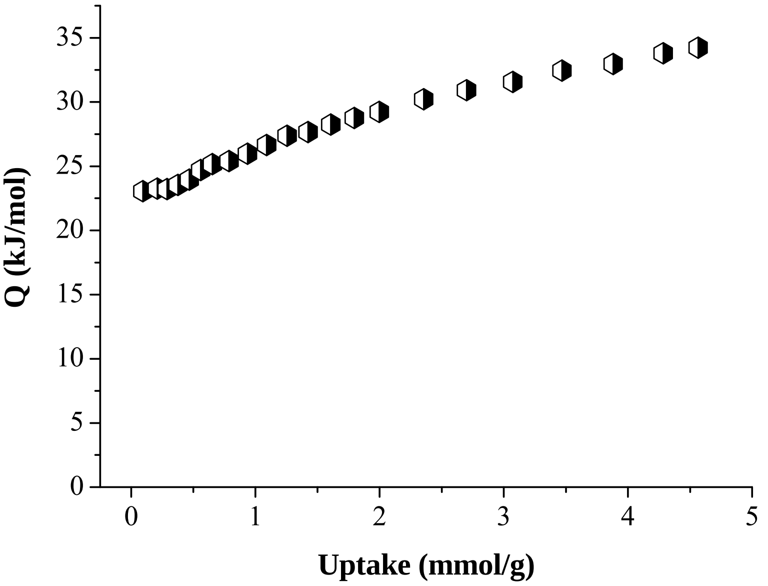

Fig. S16 Loading-dependent C_2H_2 adsorption enthalpy derived from isotherms measured at 273 and 283 K.

Table S4. Comparison of C_2H_2 storage performances of representative MOFs. The C_2H_2 uptake of the reported MOFs at 1.5 atm were obtained by simulation using the dual site Langmuir model, which fit well with the reported experimental isotherms at 0-1.0 atm.

No.	Compound	Density / g cm^{-1}	Uptake (1 atm) / mmol g^{-1}	Uptake (1.5 atm)/ mmol g^{-1}	Uptake (1 atm) / mmol cm^{-3}	Uptake (1.5 atm) / mmol cm^{-3}	*T* / K	USC / mmol g^{-1}	USC / mmol cm^{-3}	Ref.
1	HOF-1	1.275	2.83	2.879	3.61	3.673	273	0.0491	0.0625	1
2	UTSA-72	0.986	1.25	1.348	1.23	1.328	296	0.0982	0.0982	2
3	HKUST-1	0.8806	9.01	9.948	7.94	8.766	295	0.938	0.826	3
4	MOF-505	0.9257	6.65	7.65	6.16	7.089	295	1.00	0.929	
5	MOF-508	1.244	4.02	4.149	5.00	5.161	290	0.129	0.161	
6	MIL-53	0.9306	3.24	3.78	3.01	3.514	295	0.540	0.504	
7	SBMOF-2	1.191	2.91	3.017	3.46	3.589	298	0.107	0.129	4
8	Co-MOF-74	1.176	7.52	7.926	8.84	9.318	296	0.406	0.478	5
9	Mg-MOF-74	0.911	8.24	9.111	7.50	8.295	296	0.871	0.795	
10	UTSA-20	0.910	6.56	7.422	5.97	6.756	296	0.862	0.786	
11	PCN-16	0.723	7.60	9.13	5.50	6.61	296	1.53	1.11	
12	NOTT-102	0.688	5.37	6.63	3.70	4.566	296	1.26	0.866	
13	M'MOF-3a	1.023	0.125	0.1473	0.128	0.1503	296	0.0223	0.0223	
14	Cu_2TPTC-Me	0.760	9.10	11.02	6.92	8.38	298	1.92	1.46	6
15	ZJU-70a	0.876	8.35	9.46	7.31	8.279	298	1.11	0.969	7
16	UTSA-50a	1.279	4.02	4.636	5.14	5.926	296	0.616	0.786	8
17	ZJU-5	0.734	8.36	10.74	6.13	7.88	298	2.38	1.75	9
18	ZJNU-46	0.806	7.72	9.4	6.22	7.58	298	1.68	1.36	10
19	ZJNU-47	0.731	8.79	10.84	6.42	7.92	298	2.05	1.50	
20	ZJNU-48	0.825	7.91	9.83	6.53	8.12	298	1.92	1.59	
21	ZJU-30	0.727	2.29	2.335	1.67	1.701	296	0.0446	0.0313	11
22	UTSA-68	0.459	3.05	3.099	1.40	1.422	296	0.0491	0.0223	
23	ZJU-26	0.638	3.64	4.6	2.32	2.932	298	0.960	0.612	12
24	M'MOF-2a	0.997	1.91	2.133	1.91	2.133	295	0.223	0.223	13
25	Zn5(BTA)6(TDA)2	1.314	1.95	2.2	2.56	2.89	295	0.250	0.330	14
26	$Zn_4(OH)_2$(1,2,4-BTC)$_3$	1.536	2.35	2.569	3.62	3.955	295	0.219	0.335	15
27	Cu(BDC-OH)	0.91	1.96	2.121	1.78	1.927	296	0.161	0.147	16

28	UTSA-36	1.158	2.50	2.795	2.89	3.234	296	0.295	0.344	17
29	MAF-2	1.171	3.13	3.893	3.66	4.553	298	0.763	0.893	18
30	MAF-4	0.946	0.903	1.35	0.854	1.277	298	0.447	0.423	
31	MAF-7	0.912	2.26	3.37	2.03	3.04	298	1.11	1.01	
32	ZJU-40	0.698	9.21	10.15	6.43	7.084	298	0.937	0.654	19
33	NOTT-101	0.698	7.96	9.98	5.56	6.97	298	2.02	1.41	
34	SIFSIX-1-Cu	0.875	8.53	8.715	7.46	7.622	298	0.185	0.162	20
35	SIFSIX-2-Cu	0.635	5.30	6.68	3.37	4.246	298	1.38	0.876	
36	MAF-123-Cd	1.533	2.23	3.53	3.42	5.41	298	**1.30**	**1.99**	This work

Supplementary References

1 Y. He, S. Xiang and B. Chen, *J. Am. Chem. Soc.*, 2011, **133**, 14570-14573.

2 H. Alawisi, B. Li, Y. He, H. D. Arman, A. M. Asiri, H. Wang and B. Chen, *Cryst. Growth Des.*, 2014, **14**, 2522-2526.

3 S. Xiang, W. Zhou, J. M. Gallegos, Y. Liu and B. Chen, *J. Am. Chem. Soc.*, 2009, **131**, 12415-12419.

4 A. M. Plonka, X. Chen, H. Wang, R. Krishna, X. Dong, D. Banerjee, W. R. Woerner, Y. Han, J. Li and J. B. Parise, *Chem. Mater.*, 2016, **28**, 1636-1646.

5 Y. He, R. Krishna and B. Chen, *Energy Environ. Sci.*, 2012, **5**, 9107-9120.

6 T. Xia, J. Cai, H. Wang, X. Duan, Y. Cui, Y. Yang and G. Qian, *Micropor. Mesopor. Mater.*, 2015, **215**, 109-115.

7 X. Duan, C. Wu, S. Xiang, W. Zhou, T. Yildirim, Y. Cui, Y. Yang, B. Chen and G. Qian, *Inorg. Chem.*, 2015, **54**, 4377-4381.

8 H. Xu, Y. He, Z. Zhang, S. Xiang, J. Cai, Y. Cui, Y. Yang, G. Qian and B. Chen, *J. Mater. Chem. A*, 2013, **1**, 77-81.

9 X. Rao, J. Cai, J. Yu, Y. He, C. Wu, W. Zhou, T. Yildirim, B. Chen and G. Qian, *Chem. Commun.*, 2013, **49**, 6719-6721.

10 C. Song, J. Jiao, Q. Lin, H. Liu and Y. He, *Dalton Trans.*, 2016, **45**, 4563-4569.

11 G. Chang, B. Li, H. Wang, T. Hu, Z. Bao and B. Chen, *Chem. Commun.*, 2016, **52**, 3494-3496.

12 X. Duan, J. Cai, J. Yu, C. Wu, Y. Cui, Y. Yang and G. Qian, *Micropor. Mesopor. Mater.*, 2013, **181**, 99-104.

13 S. C. Xiang, Z. Zhang, C. G. Zhao, K. Hong, X. Zhao, D. R. Ding, M. H. Xie, C. D. Wu, M. C. Das, R. Gill, K. M. Thomas and B. Chen, *Nat. Commun.*, 2011, **2**, 204.

14 Z. Zhang, S. Xiang, Y.-S. Chen, S. Ma, Y. Lee, P.-B. Thomas and B. Chen, *Inorg. Chem.*, 2010, **49**, 8444-8448.

15 Z. Zhang, S. Xiang, X. Rao, Q. Zheng, F. R. Fronczek, G. Qian and B. Chen, *Chem. Commun.*, 2010, **46**, 7205-7207.

16 Z. Chen, S. Xiang, H. D. Arman, P. Li, S. Tidrow, D. Zhao and B. Chen, *Eur. J. Inorg. Chem.*, 2010, **2010**, 3745-3749.

17 M. C. Das, H. Xu, S. Xiang, Z. Zhang, H. D. Arman, G. Qian and B. Chen, *Chem. Eur. J.*, 2011, **17**, 7817-7822.

18 J. P. Zhang, A. X. Zhu, R. B. Lin, X. L. Qi and X. M. Chen, *Adv. Mater.*, 2011, **23**, 1268-1271.

19 H.-M. Wen, H. Wang, B. Li, Y. Cui, H. Wang, G. Qian and B. Chen, *Inorg. Chem.*, 2016, **55**, 7214-7218.

20 X. Cui, K. Chen, H. Xing, Q. Yang, R. Krishna, Z. Bao, H. Wu, W. Zhou, X. Dong, Y. Han, B. Li, Q. Ren, M. J. Zaworotko and B. Chen, *Science*, 2016, **353**, 141-144.
